# Investigation of hematological and biochemical parameters in small ruminants naturally infected with *Babesia ovis*

**Published:** 2012

**Authors:** Bijan Esmaeilnejad, Mousa Tavassoli, Siamak Asri-Rezaei

**Affiliations:** 1 *Department of Pathobiology, Faculty of Veterinary Medicine, Urmia University, Urmia, Iran; *; 2 *Department of Clinical Sciences, Faculty of Veterinary Medicine, Urmia University, Urmia, Iran.*

**Keywords:** Small ruminant, Babesia ovis, Hematology, Biochemical parameters

## Abstract

*Babesia ovis *plays an important role in causing anemia and kidney dysfunction in affected animals. There are few extensive studies about hematological and biochemical findings of small ruminants' babesiosis caused by *B. ovis*. The aim of this study was to evaluate the effect of babesiosis on some hematological and biochemical parameters in infected small ruminants with *B. ovis*. A total of 280 sheep and 122 goats from 40 herds were randomly examined for the presence of *B. ovis* in blood samples. Of 402 samples, 67 animals (16.7%) were positive for *B. ovis* of which 52 (18.5%) were sheep and 15 (12.2%) goats, respectively. The infected animals were divided into four subgroups according to parasitemia rates (<1%, 1%, 2%, and 3%). As a control group, 67 uninfected animals were also selected from the same farms. With increase in parasitemia rates, hemoglobin concentration (Hb), packed cell volume (PCV), red blood cells (RBCs), mean corpuscular volume (MCV) and mean corpuscular hemoglobin concentration (MCHC) significantly decreased (*P *< 0.05), while, total leukocyte count, number of lymphocyte, monocyte, neutrophil and eosinophil showed a significant increase (*P* < 0.05). Infected animals presented a significant elevation (*P *< 0.05) of total proteins and significantly lower level (*P* < 0.05) of albumin compared to non-infected animals. Significant elevation (*P *< 0.05) of BUN, creatinine, cholestrol, triglyceride, HDL and LDL level were found with parasitemia progression.

## Introduction

*Babesia ovis*, the main etiological agent of small ruminants' babesiosis, is a small form of *Babesia* parasite (1-1.5 µm in diameter), tick-transmitted and intra-erythrocytic protozoan parasite, that causes severe economic losses among sheep and goats in tropical and subtropical areas.^1^ Infection can be acute, subacute or chronic. Clinical babesiosis cases due to *B. ovis* infection are highly variable. The classic presentation is a febrile syndrome with apparent anemia and hemoglobinuria.^1^ Without treatment some animals may survive after a long convalescent period, but others may develop shock and/or renal failure leading to death.^2^ According to the previous reports, *B. ovis* is considered as a highly pathogen organism which caused ovine babesiosis in most part of Iran.^3^^,^^4^ Two species of *Babesia* are generally recognized as pathogen, *B. ovis* and *B. motasi*.^5^ Morphologic and serologic studies have been previously done.^6^^,^^7^ Razmi *et al*. determined the high prevalence of *B. ovis* in sheep and goats in North-East of Iran.^8^ Alani and Hebert described hematological and biochemical changes in splenectomized sheep experimentally infected with *B. motasi*.^9^ Several studies have been previously carried out on the histopathology of produced lesion by *B. ovis*.^10^^,^^11^^,^^12^ To our knowledge, most of the previous studies were performed on hematological and biochemical changes on experimentally infected sheep with *B. ovis*.^5^^,^^11^^,^^13^^,^^14^ The present investigation was conducted to study some hematological and serum biochemical parameters in sheep and goats which were naturally infected with *B. ovis*.

## Materials and Methods

**Source of animals and samples. **From June to September 2009, 402 small ruminants (including 280 sheep and 122 goats) from various regions of North-West, Iran (West Azerbaijan province) were randomly selected. As a control group, 67 clinically healthy animals reared under the same management and environmental conditions were also sampled. Microscopic examination of Giemsa-stained peripheral blood smears revealed *B. ovis* infection. The parasitological diagnosis was confirmed using PCR analysis. Infected animals were divided into 4 subgroups according to parasitemia rates (<1%, 1%, 2% and 3%).^1^^,^^13^

**Sampling. **Blood samples were taken from the jugular vein into vacutainers containing EDTA-K_2_ as anticoagulant for determination of hematological parameters and without EDTA-K_2_ for isolated of serum samples for biochemical analysis. The sera were separated by centrifugation at 750 *g* for 15 min and stored at –20 °C until used. Thin blood smears were prepared from ear vein of all animals.

**PCR amplification. **DNA extraction was performed according to the methods described by Clausen *et al*. with some modifications.^15^ Briefly, 125 µL of blood was added to 250 µL of lysis mixture (0.32 M sucrose, 0.01 M Tris, 0.005 M MgCl_2_, 1% Triton X-100, pH 7.5) and the mixture was centrifuged at 11600 *g* for 1 min. The pellet was washed three times with 250 µL lysis buffer by centrifugation. The supernatant was discarded and the final pellets were re-suspended in 100 µL of PCR buffer (50mM KCl, 10mM Tris-HCl (pH 8.0), 0.1% Triton X-100, and pH 8.3) containing 50 µg of proteinase K mL^-1^ and then incubated at 65 °C for 1h. Finally, the sample was boiled at 95 °C for 10 min. A pair of primers, Bbo-F 5'-TGGGCAGGACCTTGGTTCTTCT-3' and Bbo-R 5'-CCGCGTAGCGCCGGCTAAATA-3' were used to amplify a 549 bp fragment of the *ssu* rRNA gene of *B. ovis*.^16^ The final 25 µL PCR mixture contained 12.5 µL of ready to use PCR master mix (Containing dNTPs, Taq DNA polymerase and MgCl_2_, Cinagen, Iran), 2 µL of each primers (final concentration: 0.5 µM, 2 µL of extracted template DNA (≈ 10 ng) and distilled water. The PCR amplification was done using a programmable thermal cycler (Corbett Research, CP2-003, Australia). The reaction was incubated at 95 °C for 5min to denature genomic DNA and the thermal cycle reaction was programmed as follows: 45 cycles of 94 °C for 45 sec, 63 °C for 45 sec and 72 °C for 1 min. The PCR reaction was ended by a final extension at 72 °C for 10 min. The PCR products were separated by electrophoresis on 1.5% agarose gel in 0.5 times TBE buffer and visualized using ethidium bromide (1 µg mL^-1^) and UV-illuminator (BTS-20M, Japan). The 50 bp ladder (Fermentas, Germany) was used as a DNA marker in this study.

**Parasitological and hematological examination. **Parasitemia rates was recorded by microscopic examination of Giemsa-stained blood smears, as the number of piroplasm-infected erythrocytes in 100 cells.^17^ Hemoglobin (Hb) concentration, red blood cell (RBC) count, white blood cell (WBC) count, differential WBC counts and packed cell volume (PCV) were determined by automated hematology analyzer (Autolyser, Al 820, Swiss). Mean corpuscular volume (MCV) and mean corpuscular hemoglobin concentration (MCHC) were evaluated.^18^

**Serum biochemical studies. **The sera were analyzed for the measuring of serum total protein, albumin, urea, creatinine triglyceride, cholesterol, high density lipoprotein (HDL) and low density lipoprotein (LDL), using commercial test kits supplied by Pars Azmun (Tehran, Iran).

**Statistical analysis. **The results were analyzed by one-way analysis of variance (ANOVA) followed by pair-wise comparisons using the Duncan tests. Difference were considered significant when *P *< 0.05. The computer software, SPSS version 17.0 for windows was used for analysis.

## Results

PCR results revealed that 67 animals (52 sheep and 15 goats) (16.7%) were infected with *B. ovis*. The values of hematological and biochemical parameters in healthy small ruminants and those infected with *B. ovis* with different parasitemia rate are presented in Table 1 and 2, respectively.

There were significant differences in hematological indices and measured biochemical parameters between healthy and *B. ovis*-infected animals (*P *< 0.05). As the parasitemia rate increased in infected animals, a significant decrease (*P *< 0.05) was observed in RBCs, PCV, Hb, MCV, and MCHC. In contrast, with increase in parasitemia rate, significant increase (*P *< 0.05) in WBC count and concentration of serum BUN, creatinine, total protein, albumin, globulin, triglyceride, cholesterol, HDL and LDL was evident.

**Table 1:** Mean ± SEM of hematological parameters in uninfected small ruminants and those infected with *Babesia ovis* with different parasitemia rates

**Group**	**Parameters**											
	**No.**	**Parasitemia** **(%)**	**RBCs** **(10** ^6 ^ **µL** ^-1^ **)**	**PCV** **(%)**	**Hb** **(g dL** ^-1^ **)**	**MCV** **(fL)**	**MCHC** **(g dL** ^-1^ **)**	**WBC** **(10** ^3^ ** µL** ^-1^ **)**	**Neutrophil** **(10** ^3^ ** µL** ^-1^ **)**	**Lymphocyte** **(10** ^3^ ** µL** ^-1^ **)**	**Monocyte** **(10** ^3^ ** µL** ^-1^ **)**	**Eosinophil** **(10** ^3^ ** µL** ^-1^ **)**
**Control**	67	0	6.87 ±0.03^a^	31.54 ± 0.41	10.51± 0.13^a^	45.13 ± 0.40^a^	25.20 ± 0.22^a^	6.46 ± 0.12^d^	2.55 ± 0.05^e^	3.72 ± 0.06^d^	0.10 ± 0.00^d^	0.03 ±0.00^e^
**Diseased**	27	<1	6.26 ± 0.03^b^	28.00 ± 0.60^b^	9.33 ± 0.20^b^	42.17 ± 0.29^b^	22.87 ± 0.20^b^	7.66 ± 0.16^c^	2.95 ± 0.05^d^	4.41 ± 0.15^d^	0.20 ± 0.00^c^	0.05 ± 0.00^d^
	17	1	5.45 ± 0.06^c^	23.76 ± 0.65^c^	7.92 ± 0.21^c^	40.37 ± 0.40^b^	21.95 ± 0.26^bc^	9.29 ± 0.31^b^	3.72 ± 0.04c	5.24 ± 0.26^c^	0.29 ± 0.00^b^	0.05 ± 0.00^c^
	16	2	3.50 ± 0.04^d^	15.50 ± 0.10^d^	5.17 ± 0.03^d^	38.06 ± 0.40^c^	21.15 ± 0.22^c^	10.10 ± 0.13^b^	4.21 ± 0.04^b^	6.30 ±0.09^b^	0.32 ± 0.00^b^	0.06 ± 0.00^b^
	7	3	2.78 ± 0.04^e^	13.00 ± 0.21^e^	4.33 ± 0.07^e^	35.86 ± 0.50^d^	19.92 ± 0.282^d^	14.9 ± 0.218^a^	5.67 ± 0.06^a^	8.74 ± 0.07^a^	0.39 ± 0.00^a^	0.09 ± 0.00^a^

* Difference superscripted letters ( a, b, c, d, and e) denote a significant difference ( *P* < 0.05).

**Table 2:** Mean ± SEM of biochemical parameters in uninfected small ruminants and those infected with *Babesia ovis* with different parasitemia rates

**Group**	**Parameters**											
	**No.**	**Parasitemia** **(%)**	**Urea** **(mg dL** ^-1^ **)**	**Creatinine** **(mg dL** ^-1^ **)**	**Total Protein** **(g dL** ^-1^ **)**	**Albumin** **(g dL** ^-1^ **)**	**Globulin** **(g dL** ^-1^ **)**	**Cholesterol** **(mg dL** ^-1^ **)**	**Triglycerides** **(mg dL** ^-1^ **)**	**HDL** **(mg dL** ^-1^ **)**	**LDL** **(mg dL** ^-1^ **)**	**Cholesterol** **(mg dL** ^-1^ **)**
**Control**	67	0	9.32 ± 0.14^d^	0.89 ± 0.02^e^	7.10 ± 0.01^c^	3.20 ± 0.01^a^	3.90 ± 0.01^c^	6.46 ± 0.12^d^	77.08 ± 0.17^b^	55.40 ± 0.33^e^	5.07 ± 0.94^e^	19.40 ± 0.16^e^
**Diseased**	27	<1	9.80 ± 0.15^d^	1.07 ± 0.03^d^	7.97 ± 0.08^b^	2.46 ± 0.10^b^	4.70 ± 0.19^b^	7.66 ± 0.16^c^	78.70 ± 0.38^a^	56.70 ± 0.37^ d^	5.80 ± 0.16^d^	20.00 ± 0.29^d^
	17	1	10.71 ± 0.25^c^	1.34 ± 0.05^c^	8.01 ± 0.10^b^	2.42 ± 0.12^b^	5.80 ± 0.18^b^	9.29 ± 0.31^b^	79.68 ± 0.50^a^	58.40 ± 0.48^c^	6.20 ± 0.20^c^	21.50 ± 0.37^c^
	16	2	13.70 ± 0.22^b^	2.10 ± 0.03^b^	8.19 ± 0.09^b^	2.20 ± 0.10^b^	6.60 ± 0.30^b^	10.10 ± 0.13^b^	81.00 ± 0.51^a^	59.90 ± 0.47^b^	6.40 ± 0.21^b^	22.00 ± 0.40^b^
	7	3	17.80 ± 0.38^a^	2.70 ± 0.04^a^	8.72 ± 0.08^a^	1.55 ± 0.12^c^	7.60 ± 0.09^a^	14.9 ± 0.218^a^	81.9 ± 0.62^a^	61.5 ± 0.64^a^	6.58 ± 0.16^a^	22.90 ± 0.51^a^

* Difference superscripted letters ( a, b, c, d, and e) denote a significant difference ( *P* < 0.05).

## Discussion

According to the present study, different parasitemia rates were observed in the infected animals. These observations were in accordance with the findings by Sevinc *et al.*, Razmi *et al.* and Aktas *et al*.^1^^,^^8^^,^^19^ Decrease in RBCs, PCV and Hb level in infected animals were significantly lower than healthy animals (*P* < 0.05). These results were consistent with previous findings by Voyvoda *et al.* and Hadadazadeh *et al.*^20^^,^^21^ In addition, decline in PCV, Hb content and RBCs observed in other studies that was previously performed on clinicophathological changes that induced by *B. equi* and *B. gibsoni*.^22^^,^^23^ The present anemia may be attributed to immunomediated phenomena by autoantibodies directed against component of membrane of infected and uninfected erythrocytes,^24^ production of toxic hemolytic factors of the parasite,^25^ mechanical damage by trophozoite intra-erythrocytic binary fission,^26^ erythrophagocytosis and through of release vasoactive molecules such as kallikrein.^2^^,^^27^ Concerning the erythrocyte indices, with parasitemia rates progression, a significant decrease was observed in MCV and MCHC. As parasitemia increased, a depletion in MCV and MCHC was evident that indicated microcytic-hypochromic anemia. The result was in accordance with reports by Rahbari *et al.*,^5^ and Rubino *et al.*^24^ Moreover, Zobba *et al.* reported microcytic-hypochromic anemia in horse infected with *B. equi*.^26^

On the other hand, polychromatophilic erythrocytes in blood smears pointed out a hemolytic anemia. Reduction in MCV level may be due to two reasons. First, decrease in PCV level may be attributed to the dilution of blood and subsequently MCV could decrease.^2^ Second, the most common abnormality of erythrocytes parameters is anisocytosis (spherocytosis) which was detected in 34 out of 52 (65.3%) of infected animals and in reference to the value of MCV which was below the normal values associate with spherocytosis.^29^ Polychromatophilic erythrocytes (Synonymous reticulocytes) have a deficient component of hemoglobin, therefore, the MCHC decreases in ovine/caprine bebesiosis.^27^ The leukogram revealed a significant increase in WBCs while parasitemia rates increased. The observed leukocytosis in infected animals in this study, are consistent with findings by other researchers.^20^^,^^23^^,^^28^^,^^30^ However, these results differ from findings by Moreau *et al*.,^33^ Rahbari *et al.*,^5^ and Hadadzadeh *et al.*^21^ The difference probably resulted from infection of animals with different subspecies of *B. ovis* and also seemed to be due to the extended tissue damage. Furlanello *et al.* reported that leukocytosis occurred due to maturation of neutrophil and lymphocyte.^31^ The observed eosinophilia was due to the sensitivity to the foreign protein of a parasite which may be a part of an immune phenomenon.^32^ Similar to the present study, monocytosis was reported by Wright *et al.*,^34^ and Gazzinelli *et al*.^35^ Monocytes are the host cells stimulated and invaded by *B. bovis* in vitro.^36^^,^^37^ In addition, macrophage activation is known to occur during babesiosis and a protective role has been documented for macrophages during infection with several *Babesia* species.^24^ Hemoparasite-activated macrophages release proinflamatory cytokines, including interlukin-1 (IL-1), interlukin-12 (IL-12) and tumor necrosis factor (TNF).^38^ Interlukin-1 causes the proliferation of lymphocytes and T helper cells activated by IL-12 produces gamma interferon (IFN-γ). The latter and TNF are also important for activating of blood mononuclear cells (Lymphocytosis and monocytosis) and polymorphonuclear cells (neutrophilia).^37^^,^^39^ In addition, neutrophilia attributes to chemotactic effect of TNF on neutrophil. Neutrophils are also the chemical mediators of acute inflammation.^24^^,^^40^ In the current study, as parasitemia increased, a significant elevation was evident in BUN and creatinine level. The results are in consistent with findings by other researchers.^5^^,^^12^^,^^41^ It is known that renal involvement occurs in *B. ovis* infection.^5^^,^^11^ Observed elevation in BUN and creatinine level might have resulted from kidney dysfunction,^5^ muscle catabolism,^12^ and colonization of *B. ovis* in the renal blood circulation.^11^ It is suggested that in ovine babesiosis; many potential factors leading to impairment renal function, e.g., acute diffuse proliferative glomerulitis, acute glomerular hemorrhage, presence of thrombi, congestion and stasis in glomerular capillaries, acute glomerular hemorrhage and acute tubular necrosis.^5^^,^^11^ Main observed histopathological changes in kidneys in naturally acquired *B. canis* infection were vacuolar-hydropic degeneration, necrosis, detachment of renal tubular epithelial cells in proximal convoluted tubules and hemoglobin casts.^42^ Moreover; hypoxia appears to be more important than hemoglobinuria in damaging the kidney of experimentally and naturally *Babesia*-infected dogs. Systemic hypotension leading to vasoconstriction in the kidney might be the most important case of renal hypoxia in *B. canis* infection, but anemia may also contribute to inadequate oxygenation.^43^ In addition to, both renal infarction and disseminated intravascular coagulation (DIC) were reported in experimentally infected cattle with *B. ovis*.^11^ Finally, it seems that elevation in BUN and creatinine level ascribes to kidney malfunctions in infected small ruminants.

To our knowledge, the current study is the first report of lipids and protein profile in small ruminants infected with *B. ovis*. The observed hypoalbuminemia in current study, is in agreement with those reported earlier.^23^^,^^44^ Reduction of albumin level probably corresponds to disturbance in liver function, urinary loss of albumin associated with renal failure (proteinuria) and anorexia in relation to high rise of body temperature. Similar results have been reported previously.^45^^-^^47^ Concerning the total protein and globulins level, an increase were observed. The results are in accordance with findings by other investigators .^23^^,^^24^^,^^46^ The observed hyperpoteinemia can be attributed to an increase in the globulin concentration in response to parasitic antigen and released hemoglobin from destructed erythrocytes.^44^^,^^46^ reportedly, significant correlation were observed between albumin and cholesterol (positive), albumin and triglycerides (positive).^46^ With an increase in parasitemia rates, reduction in cholesterol and triglycerides concentration was expectable. The slight lipidemia can be ascribed to liver compensatory reaction to the loss of protein, including HDL and LDL.^46^ According to Rees and Schoman, insulin concentration in dogs with babesioisis (*B. canis* *rossi*) is low.^48^ On the other hand, the metabolism of adipose tissue (Lipogenesis) is strictly related to insulin. Consequently, reduced-level of insulin during babesiosis seems other reason for less observed elevation in cholesterol and triglycerides content. We do not have clear explanation for the increase in HDL and LDL levels. Age, sex, breed, nutrition quality and different stage of pregnancy may affect lipoproteins' concentration. 

In conclusion, further studies are needed to precisely define the pathophysiology of small ruminants' babesiosis and serum protein response in *B. ovis* infection. 
